# Inhibition of NFκB by the natural product Withaferin A in cellular models of Cystic Fibrosis inflammation

**DOI:** 10.1186/1476-9255-6-15

**Published:** 2009-05-13

**Authors:** Rangan Maitra, Melissa A Porter, Shan Huang, Brian P Gilmour

**Affiliations:** 1 Center for Organic and Medicinal Chemistry, The Research Triangle Institute, Research Triangle Park, NC 27709, USA; 2 Department of Chemistry, Duke University, Box 90354, Durham, NC 27708-0354, USA

## Abstract

Cystic Fibrosis (CF) is one of the most common autosomal genetic disorders in humans. This disease is caused by mutations within a single gene, coding for the cystic fibrosis transmembrane conductance regulator (CFTR) protein. The phenotypic hallmark of CF is chronic lung infection and associated inflammation from opportunistic microbes such as *Pseudomonas aeruginosa *(PA), *Haemophilus influenzae*, and *Staphylococcus aureus*. This eventually leads to deterioration of lung function and death in most CF patients. Unfortunately, there is no approved therapy for correcting the genetic defect causal to the disease. Hence, controlling inflammation and infection in CF patients are critical to disease management. Accordingly, anti-inflammatory agents and antibiotics are used to manage chronic inflammation and infection in CF patients. However, most of the anti-inflammatory agents in CF have severe limitations due to adverse side effects, and resistance to antibiotics is becoming an even more prominent problem. Thus, new agents that can be used to control chronic inflammation in CF are needed in the absence of a cure for the disease. Activation of the transcription factor NFκB through Toll-like receptors (TLR) following bacterial infection is principally involved in regulating lung inflammation in CF. NFκB regulates the transcription of several genes that are involved in inflammation, anti-apoptosis and anti-microbial activity, and hyper-activation of this transcription factor leads to a potent inflammatory response. Thus, NFκB is a potential anti-inflammatory drug target in CF. Screening of several compounds from natural sources in an *in vitro *model of CF-related inflammation wherein NFκB is activated by filtrates of a clinically isolated strain of PA (PAF) led us to Withaferin A (WFA), a steroidal lactone from the plant *Withania Somnifera L. Dunal*. Our data demonstrate that WFA blocks PAF-induced activation of NFκB as determined using reporter assays, IL-8 measurements and high-content fluorescent imaging of NFκB subunit p65 translocation. Since the airways of CF patients can be specifically targeted for delivery of therapeutics, we propose that WFA should be further studied as an anti-inflammatory agent in models of CF related inflammation mediated by NFκB.

## Findings

Cystic Fibrosis (CF) is one of the most common lethal autosomal recessive diseases in humans. It is caused by mutations within a single gene, coding for the cystic fibrosis transmembrane conductance regulator (CFTR) protein (reviewed in [1]). Loss of lung function causes over 90% of all CF deaths [2,3], which is brought about by chronic bacterial infections involving drug-resistant pathogenic strains of *Pseudomonas aeruginosa (PA)*, *Haemophilus influenzae *and *Staphylococcus aureus *[3-5] among others. Chronic and uncontrolled stimulation of cellular signaling by bacterial products through toll-like receptors (TLRs) lead to hyper-activation of the transcription factor NFκB and over-expression of a number of pro-inflammatory cytokines [6-8]. Consequently, an overwhelming number of neutrophils and macrophages are attracted to the site of infection and these cells release proteases and other agents that cause structural damage to the airways. Anti-inflammatory agents are used to manage lung inflammation in CF, but have adverse effects [9] that limit their use. Thus, there is a need to identify drugs with limited toxicity to treat lung inflammation in CF [10].

Screening of natural products with purported anti-inflammatory activity led us to Withaferin A (WFA), a steroidal lactone isolated from the herb *Withania somnifera *(also known as Indian Ginseng and Ashwagandha), which is widely used in traditional Indian medicine as an anti-inflammatory agent [11]. Recent reports indicate that this natural product is an inhibitor of NFκB activity [12,13]. The overall goal of this study was to characterize the effect of this compound on NFκB in cellular models of CF-related inflammation. In our studies, filtrates of PA isolated from a CF patient were used. This is an established method to experimentally induce inflammation in the field of CF research and is relevant to airway inflammation noted in patients [14,15]. Inflammation in CF is caused by a complex mixture of bacterial products including secreted toxins, lipoproteins, lipopolysaccharides and bacterial DNA [16]. The filtrates used in our studies isolated from post log-phase cultures of PA contain many of these harmful agents. These products differentially activate various TLRs expressed in airway epithelial cells and ultimately increase expression of pro-inflammatory genes regulated by NFκB [17].

Unless specified, all reagents were purchased from Sigma Aldrich (St. Louis Missouri). The KKLEB immortalized CF airway cell line homozygous for ΔF508 mutation and the CF 14 clinically isolated mucoid strain of PA (originally from the laboratory of Dr. M.C. Wolfgang, University of North Carolina) were donated by Dr. S. Randell (University of North Carolina) [15]. HEK 293 cells were obtained from ATCC (Manassas, VA). Cells were maintained in DMEM/F12 medium with 10% fetal bovine serum and antibiotics. A NFκB reporter plasmid was constructed as follows: Complementary oligonucleotides bearing NFκB consensus DNA-binding sequence (5'-gctagc tgg gga ctt tcc gct ggg gac ttt ccg ctg ggg act ttc cgc tgg gga ctt tcc gct ggg gac ttt ccg c aagctt-3') were synthesized with flanking NheI and HindIII sites (underlined), annealed and introduced into the pGL4.26 (luc2/minP/Hygro) vector (Promega, Madison, WI). The construct was linearized with Bsu36I and transfected into HEK293 cells using Fugene HD (Roche Diagnostics, Indianapolis, IN). Clonal cell-lines stably expressing the construct were identified following selection in Hygromycin-containing media and tested for NFκB-mediated induction of luciferase reporter activity using recombinant TNF-α and filtrates of a clinically isolated mucoid strain of PA (PAF) from a CF patient (data not shown). A stable cell line designated HEK293/NFκB-luc was used for the reported experiments. For transient transfection assays, KKLEB cells were batch transfected using Fugene HD reagent in suspension and subsequently plated out into 24-well plates. This approach nullified the need to use a second reporter gene for data normalization. Unless otherwise noted, cells were allowed to incubate overnight and then induced with PAF for 24 hr in serum-containing media. Typically, cells were pre-incubated with WFA (Chromadex, Santa Ana, CA) for 2 hr and then stimulated with PAF that were prepared essentially as described previously [15]. Briefly, the clinically isolated mucoid strain of PA was grown for 72 hr in LB media. The supernatant from the culture was removed by centrifugation, boiled for 10 min to inactivate proteolytic activity, aliquoted, and stored at -80°C. Luciferase assays were conducted using a kit obtained from Promega in a TECAN plate-reader. Quantification of IL-8 in media was performed using a commercially available sandwich ELISA kit (Biolegend Inc., San Diego, CA). For the NFκB subunit p65 translocation studies using immunofluorescence microscopy, KKLEB cells were seeded in black optical-bottom 96-well plates and treated as described above with PAF and WFA. Following treatment, cells were fixed with 3.7% formaldehyde in PBS, and fluorescently labeled using a commercially available kit (NFκB activation HCS kit, Thermo Scientific, Waltham, MA). Fluorescent images were acquired at 20× magnification using a Discovery 1 automated fluorescent microscope (MDS Analytical Devices) with filter sets appropriate for FITC (for p65 detection) and DAPI (nuclear stain). Six images were analyzed per group resulting in analysis of roughly 300 cells per treatment. Nuclear translocation of p65 was measured using the enhanced translocation module from the Metaxpress image analysis software provided with the instrument. Input settings delineating cell "compartment" (nucleus) and "regions for measurement" (cytoplasm) were entered as follows: Compartment-width = 10 μm, intensity above background = 200 gray levels, minimum area = 5 μm^2^, and maximum area = 1000 μm^2^. Regions for Measurement (RFM) were entered as follows: Inner region distance from edge = 1 μm, outer region distance from edge = 1 μm, outer region width = 6 μm, background correction = none. Cells were scored as positive for nuclear translocation of p65 if the correlation coefficient was 0.75 or greater. Cell viability was monitored using the Cell-Titer Glo Luminescent Cell Viability Assay (Promega Corporation) following the manufacturer's suggestion. All concentrations used for our studies were non-cytotoxic to the cells (data not shown) under the experimental conditions.

**Figure 1:**
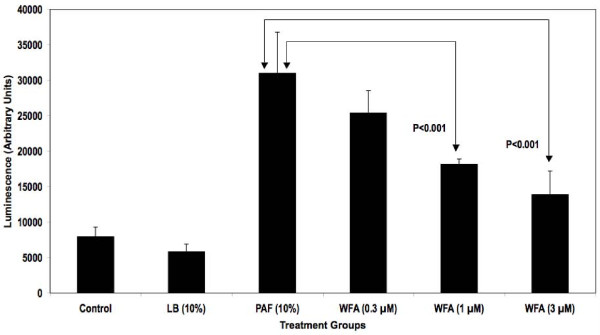
**WFA pre-treatment inhibits NFκB reporter activity in HEK293/NFκB-luc cells stimulated with PAF**. Cells were plated and treated as described in the "Findings" section. Treatment groups were as follows: Cells treated with media only (Control), cells treated with media containing 10% LB Broth (LB), cells treated with 10% PAF (PAF), cells pre-treated with various concentrations of WFA followed by stimulation with 10% PAF (WFA, 0.3, 1 and 3 μM). Data from luciferase reporter assays are reported as averaged arbitrary mean luminescence + standard deviation from 6 samples per group. Withaferin pre-treated samples were compared to PAF-treated samples using Student's t-test. Statistical significance was noted at 1 and 3 μM concentrations in WFA pre-treated cells compared to PAF only cells as indicated.

**Figure 2:**
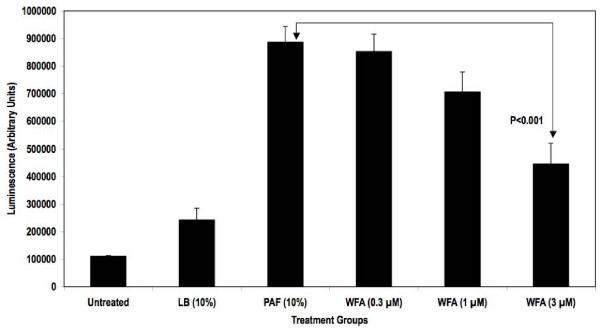
**WFA pre-treatment inhibits NFκB reporter activity in transiently transfected KKLEB cells stimulated with PAF**. Cells were plated and treated as described in the "Findings" section. Treatment groups were as follows: Cells with media only (Untreated), cells treated with media containing 10% LB Broth (LB), cells treated with 10% PAF (PAF), cells pre-treated with various concentrations of WFA followed by stimulation with 10% PAF (WFA, 0.3, 1 and 3 μM). Data from luciferase reporter assays are reported as averaged arbitrary mean luminescence + standard deviation from 3 samples per group. Withaferin pre-treated samples were compared to PAF-treated samples using Student's t-test. Statistical significance was noted at 3 μM concentrations in WFA pre-treated cells compared to PAF only cells as indicated.

**Figure 3:**
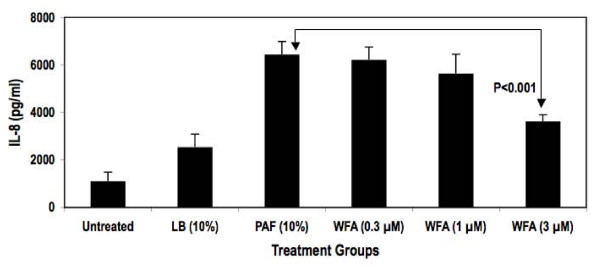
**WFA pre-treatment inhibits IL-8 secretion from KKLEB cells stimulated with PAF**. Cells were plated and treated as described in the "Findings" section. Treatment groups were as follows: Cells with media only (Untreated), cells treated with media containing 10% LB Broth (LB), cells treated with 10% PAF (PAF), cells pre-treated with various concentrations of WFA followed by stimulation with 10% PAF (WFA, 0.3, 1 and 3 μM). After 24 hr of incubation, media from each sample was collected and analyzed for IL-8 secretion using ELISA. Concentration of IL-8 was calculated by fitting the optical density of each sample to a standard curve prepared with recombinant IL-8 using linear regression. Data are reported as mean IL-8 secretion + standard deviation averaged from 3 samples per group. Withaferin pre-treated samples were compared to PAF-treated samples using Student's t-test. Statistical significance was noted at 3 μM as indicated.

An NFκB-responsive luciferase reporter construct was used to test the hypothesis that PAF-stimulated NFκB activity diminished upon treatment with WFA. As demonstrated in Figure 1, WFA pre-treatment significantly inhibited NFκB luciferase reporter activity stimulated by PAF in a concentration-dependent fashion by as much as 70% in HEK293/NFκB-luc cells. Past reports indicate that HEK293 cells express certain TLR isoforms that were activated by bacterial factors present in PAF used for our studies leading to NFκB reporter activity [18,19]. Further testing of WFA was conducted in a more relevant *in vitro *model of CF inflammation. In this model, the immortalized CF epithelial cell line KKLEB harboring the most common and severe CFTR mutation (ΔF508, noted in > 90% CF patients) was stimulated with PAF and NFκB activity was subsequently measured using three different methods. First, a transiently transfected luciferase reporter was used to monitor NFκB activation by PAF with or without WFA pre-treatment. As reported in Figure 2, WFA pre-treatment significantly diminished luciferase activity compared to control groups in a concentration-dependent fashion by ~70% in the KKLEB cells. Inflammation in CF is regulated largely by activation of NFκB and transcription of pro-inflammatory genes regulated by this transcription factor [16,20]. Therefore, we examined the effect of WFA pre-treatment on IL-8 secretion upon challenge with 10% PAF in KKLEB cells using ELISA. Inhibition of PAF-stimulated secretion of IL-8 protein was noted upon pre-treatment with WFA (Figure 3) by ~50% in KKLEB cells in agreement with reporter assays.

**Figure 4:**
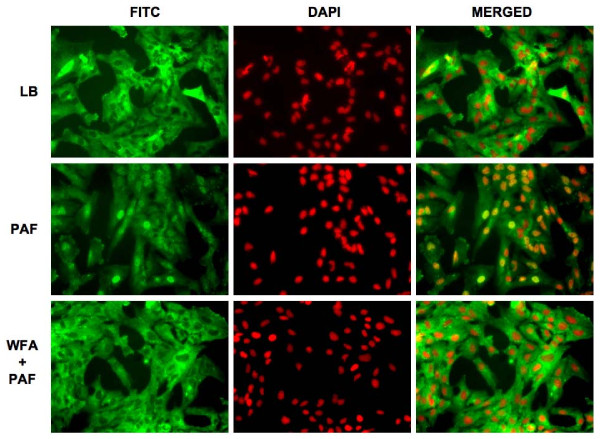
**WFA pre-treatment inhibits nuclear translocation of NFκB subunit p65 in KKLEB cells stimulated with PAF**. Cells were plated out, treated and immunostained for fluorescence microscopy in optical-bottom 96-well plates as described in "Findings". Images of fixed cells were captured using a Discovery 1 system at 20× magnification under identical conditions across all sample wells. The images were not digitally processed prior to analyses. Six images were analyzed per group. Representative images are shown. For presentation, images were imported into Image J (NIH, Bethesda, MD) and pseudocolored. A FITC-conjugated antibody was used to detect p65 (left set of columns, pseudocolered green) and DAPI was used to delineate the nuclei of each cell (middle set of columns, pseudocolored red). Merged images are shown in the right column. Images are grouped as follows (top to bottom rows): Control cells treated with media containing 10% LB Broth (LB), cells treated with 10% PAF (PAF), cells pre-treated with WFA (3 μM) followed by stimulation with 10% PAF (WFA + PAF). Translocation of p65 to the nucleus is clearly noted upon treatment with PAF. Translocation of p65 is inhibited by WFA pre-treatment leading to decreased FITC-associated fluorescence in nuclei of KKLEB cells as demonstrated in the bottom row.

**Figure 5:**
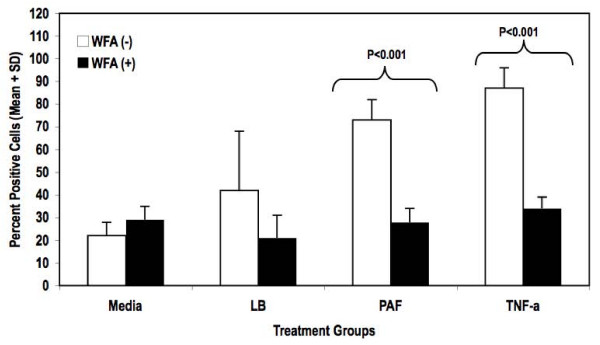
**Quantification of p65 translocation upon PAF stimulation and inhibition by WFA**. Quantification of p65 translocation into nuclei of KKLEB cells under various test conditions was performed as described in "Findings". Treatment groups are as follows: Cells with media only (Media), cells treated with media containing LB Broth (LB), cells treated with 10% PAF (PAF), cells treated with 25 ng/ml TNF-α (TNF-α). All groups were either pre-treated with vehicle (WFA (-)) or with 3 μM WFA (WFA (+)). Data are reported as percent mean + standard deviation of cells scored as positive for p65 translocation. The treated sample groups were statistically different from untreated sample groups as determined using two-way ANOVA (p < 0.05). Pair-wise comparisons between groups were performed using Bonferroni post-tests. Statistical significance (if applicable) was noted on the figure. Statistical tests were performed using Prism software (GraphPad, La Jolla, CA).

The term NFκB commonly refers to a p50–p65 heterodimer, which is the major Rel/NFκB complex in most cells [21,22]. In order to further characterize inhibition of NFκB by WFA, we investigated the effect of WFA on translocation of the NFκB subunit p65 upon stimulation with PAF using high-content immunofluorescence imaging (Figure 4). WFA pre-treatment clearly inhibited p65 translocation into the nucleus in KKLEB cells (Figure 4, bottom panel). Quantification of the images indicated that translocation of p65 was inhibited by > 80% in cells pre-treated with WFA (Figure 5).

CF as a disease is largely limited to Caucasians. According to a recent report, ~30% of patients suffering from progressive medical conditions including CF use complementary and alternative approaches to supplement conventional therapies [23]. We are intrigued by this finding, as there are many promising anti-inflammatory and anti-bacterial ethnopharmacological agents that have not been adequately studied in the context of diseases that are atypical in native populations, such as CF, where they may provide a benefit. Thus, our long-term goal is to develop strategies and platforms to test such agents in CF and other diseases. Our studies with WFA demonstrate the potential of natural products in preventing inflammation in CF mediated by NFκB. WFA itself is toxic to cells at high concentrations [24] but not at the concentrations used in this study. However, other less toxic withanolides [25] from *W. somnifera *may be useful as early leads to treat inflammation in CF. Alternatively, structure activity studies of WFA using medicinal chemistry may lead to compounds that inhibit NFκB activity without undesirable side effects. Further, targeted delivery of anti-NFκB agents to the airways of CF patients is possible using inhaled aerosols [26], which would restrict their effects largely to target tissues where this transcription factor is reported to be hyper-activated. We propose to explore some of these possibilities in future studies.

## Competing interests

The authors declare that they have no competing interests.

## Authors' contributions

RM developed the idea, secured funding, conducted certain *in vitro *experiments and was responsible for scientific and budgetary management. BG developed the molecular biology and HCS methods. MP conducted the imaging studies and provided technical assistance throughout the project. SH performed the molecular cloning and characterization of the cells used in the study.
